# Na/K-ATPase assay in the intact mice lung subjected to perfusion

**DOI:** 10.1186/1756-0500-7-798

**Published:** 2014-11-15

**Authors:** Cassiano Felippe Gonçalves-de-Albuquerque, Patrícia Burth, Adriana Ribeiro Silva, Isabel Matos Medeiros de Moraes, Flora Magno Jesus de Oliveira, Ricardo Erthal Santelli, Aline Soares Freire, Mauricio Younes-Ibrahim, Hugo Caire de Castro-Faria-Neto, Mauro Velho de Castro-Faria

**Affiliations:** Instituto Oswaldo Cruz, Laboratório de Imunofarmacologia, FIOCRUZ, 21040-900 Av Brasil 4365, Pavilhão Ozório de Almeida, Rio de Janeiro, RJ Brazil; Departamento de Biologia Celular e Molecular, Instituto de Biologia, Universidade Federal Fluminens, Niterói, RJ Brazil; Departamento de Química Analítica, Universidade Federal do Rio de Janeiro, Rio de Janeiro, RJ Brazil; Departamento de Medicina Interna, Faculdade de Ciências Médicas, Universidade do Estado do Rio de Janeiro, Rio de Janeiro, RJ Brazil

**Keywords:** Lung, Na/K-ATPase, Inductively coupled plasma optical emission spectrometer

## Abstract

**Background:**

Among the characteristics of acute respiratory distress syndrome (ARDS) is edema formation and its resolution depends on pneumocyte Na/K-ATPase activity. Increased concentration of oleic acid (OA) in plasma induces lung injury by targeting Na/K-ATPase and, thus, interfering in sodium transport.

**Findings:**

Presently, we adapted a radioactivity-free assay to detect Na/K-ATPase activity in perfused lung mice, comparing the inhibitory effect of ouabain and OA. We managed to perfuse only the lung, avoiding the systemic loss of rubidium. Rb^+^ incorporation into lung was measured by inductively coupled plasma optical emission spectrometry (ICP OES) technique, after lung tissue digestion. Na/K-ATPase activity was the difference between Rb^+^ incorporation with or without ouabain. Lung Na/K-ATPase was completely inhibited by perfusion with ouabain. However, OA caused a partial inhibition.

**Conclusions:**

In the present work the amount of incorporated Rb^+^ was greater than seen in our previous report, showing that the present technique is trustworthy. This new proposed assay may allow researchers to study the importance of Na/K-ATPase activity in lung pathophysiology.

## Background

The first description of acute respiratory distress syndrome (ARDS) appeared in 1967 [1]. One of the hallmarks of ARDS is increased alveolar capillary permeability leading to an interstitial and alveolar edema [2, 3]. The resolution of pulmonary edema and of lung inflammation are relevant factors for ARDS outcome [4]. Fluid management is one of the most important measures impacting ARDS, and a dynamic monitoring of the lung fluid balance seems to influence the clinical prognosis [5]. The removal of alveolar edema depends on the transport of salt and water across the alveolar epithelium into the lung interstitium via the basolaterally located Na/K-ATPase [6–8], which in turn drives the passive water flow toward the capillary net through the aquaporins [8].

Na/K-ATPase assay methods based on the incorporation of Rb^+^ by cultured cells [9], as well as the measurement of Rb^+^ efflux in the study of ion channels [10] have been described. The use of the non-radioactive Rb^+^ isotope as a K^+^ substitute avoids the manipulation of radioactive material. Rubidium, an element not found in biological systems, may be also measured by the atomic absorption spectrometry method.

We considered important to test the effect of the Na/K-ATPase inhibitors in a perfused lung model, which would allow the evaluation of the efficacy of these inhibitors in the intact organ.

As a first step, we propose a method to assay the Na/K-ATPase activity in absence or presence of ouabain and oleic acid (OA). In a previous report, we had developed a method for measuring Na/K-ATPase activity in intact guinea pig livers, based on the measurement of the uptake of non-radioactive Rb^+^ during *in situ* perfusion in the absence of K^+^ in the perfusion medium [11]. Differently now, we compared Na/K-ATPase inhibition by ouabain, a classical and specific Na/K-ATPase inhibitor, with OA, also known to induce lung injury in mice lung [12–14]. OA targets this enzyme *in vitro* [15] and *in vivo* leading to lung injury [16, 17]. OA levels are elevated in pathological conditions such as severe leptospirosis [18] and sepsis [19], diseases evolving to ARDS.

In conclusion, in the present report we compared the inhibition caused by ouabain to that produced by OA in an *in vivo* perfused lung mice model.

## Findings

**Figure 1:**
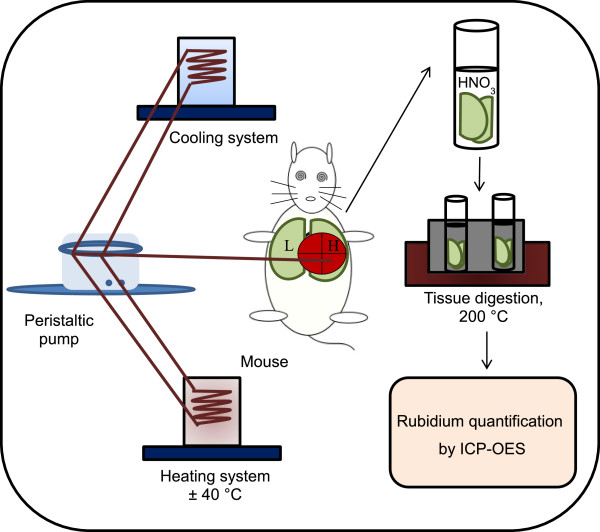
**Experimental scheme: H – heart, L – lung; ICP-OES - Inductively Coupled Plasma Optical Emission Spectrometer.**

**Figure 2:**
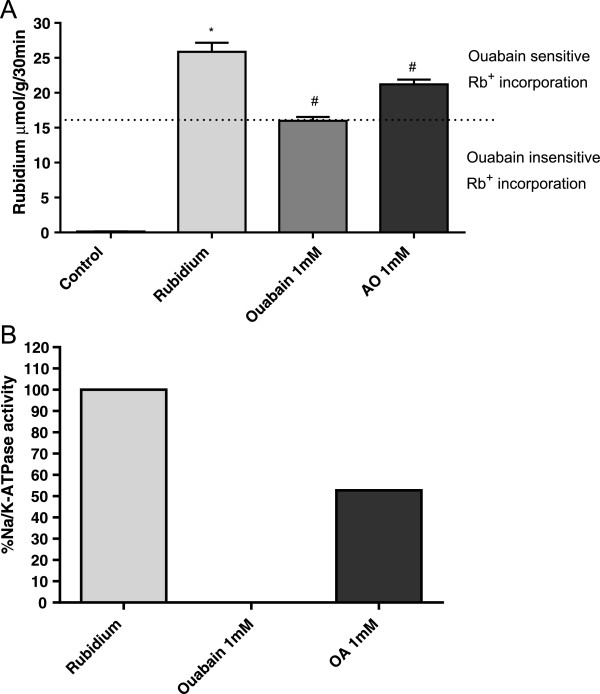
**Inhibition of Rb** ^**+**^ **incorporation in lung tissue by ouabain and OA. (A)** Mice were divided into four groups. Lungs of mice from the control group were perfused with Rb free-Hank’s. Mice lungs from the second group were perfused with KCl free-Hank’s solution. The third group was perfused with KCl free-Hank’s plus ouabain and mice lungs from the 4th group were perfused with KCl free-Hank’s plus tris-oleate. Rb^+^ incorporation in lungs was measured after 15 min by ICP-OES in digested lung tissues. Results are expressed in μmol Rb^+^ incorporated per h per g of wet tissue ± SEM of 5 to 13 animals in each group. **(B)** Calculated percent of ouabain sensitive inhibition of Na/K-ATPase based on data from Figure 2A (the difference between Rb^+^ incorporation in absence and in presence of ouabain was considered as 100% enzyme activity). Ouabain insensitive Rb^+^ incorporation represents the amount of Rb^+^ into the tissue that enters through potassium channels and passive diffusion. The experiment was repeated twice. *P <0.002, compared to controls.

Initially we defined the ideal perfusion flow rate based on our previous work with guinea pig liver, in which the ideal flow rate was 3 mL/min. We also defined a short rinsing period (5 min) because it was proved effective for efficient rinsing [11]. Besides, *in vivo* Rb^+^ quantification in mice lung showed a decrease of lung Na/K-ATPase activity observed 30 min after a single shot of KCl free-Hank’s containing ouabain or OA [16]. In the present work, we choose the time of 15 min of perfusion for two reasons: i) in 15 min, ouabain inhibition could already be detected; ii) mice lung is smaller than guinea pig liver, being quickly perfused with the same flow rate. A scheme of *in situ* perfusion is showed in Figure 1. Na/K ATPase activity was almost completely blocked by 1 mM ouabain in guinea pig liver [11]. Therefore, in the present work, we also used ouabain 1 mM to show a marked inhibition of rubidium incorporation in mice lung (Figure 2A).

Oleic acid is used to induce lung injury [17] and it is associated to increased mortality and complication in patients with high levels of this free fatty acid in blood. OA also targets Na/K-ATPase [20–22]. Previous results from our group in *in vitro* experiments showed Na/K-ATPase inhibition near 58% by OA [21]. Our present data show that the Na/K-ATPase inhibition by OA was approximately the half as compared to ouabain, as assessed by our Rb^+^ incorporation assay (Figure 2B).

## Discussion

After Rb^+^ incorporation, a short rinse with saline appeared to remove the perfusion medium from the vascular space efficiently. In guinea pig liver it would take less than one minute to change all the intravascular liquid at a perfusion rate of 3 ml/min. Assuming that mice lung is a lot less heavy (weighing 200 mg – 350 mg), most of Rb^+^ would be removed from lung vascular system after 5 min.

Our previous work with liver showed a direct proportionality between ouabain-sensitive Rb^+^ uptake and incubation time up to 60 min, evidencing that ATP concentration in the tissue remained at acceptable levels for the enzyme function [11]. We perfused during a shorter period and it is worth to mention that the enzyme was not short of ATP. We assume that the ouabain dose completely blocked the rubidium incorporation that depends on ATPase because of Na/K-ATPase inhibition. It is important to note that data from *in vivo* experiments using higher ouabain concentrations killed the majority of the animals [16]. The ouabain-insensitive Rb^+^ measurement can be assigned not only to an incomplete tissue-washing procedure but also to a passive Rb^+^ incorporation through K^+^ channels.

The main advantage of the present modified method is that it increases the sensitivity for Rb^+^ quantification: rubidium incorporation was about 20 times higher than seen in our previous work [16].

Inflammation induced by OA intravenous administration resembles ARDS in many morphological, histological and physiological aspects [14]. In this regard, ARDS patients or at-risk patients who subsequently develop ARDS have increased plasma OA concentrations [23]. Sepsis patients at risk to develop ARDS [24] present markedly increased plasma OA levels compared to healthy volunteers [25]. In most ARDS patients, the edema resolution and Na/K-ATPase activity are impaired and patients having reduced ability to clear edema have increased mortality [6, 26, 27], suggesting that Na/K-ATPase is an important player in the pathophysiology of ARDS [28]. Because of the great importance of OA in several pathologies, we decide to compare its effect on Na/K-ATPase activity with the lung Na/K-ATPase inhibition caused by ouabain. Our previous report showed that the ouabain and OA had similar *in vivo* effect on the Na/K-ATPase [16], probably because we used higher OA doses. However, Na/K-ATPase is not the sole target for OA. Since ouabain was more effective in lung Na/K-ATPase inhibition than OA, we suggest that OA should have additional targets in cell membranes, such as fatty acid receptor and fatty acid transporters [29–33].

It is worth to point out that, comparing with our previous communication [16], lung perfusion allows more Rb^+^ to be incorporated in lung tissue. In the present communication, as compared to our previous work, we noted a higher sensitivity in Rb^+^ detection allowing differential detection between oleic acid and ouabain on Rb^+^ incorporation. The fact that OA can cause lung injury with alveoli disruption has been well characterized in animal models [34]. This could explain why lung injury induced by OA is more severe than that one induced by ouabain. Nevertheless, lung injury has an important correlation with Na/K-ATPase inhibition [16].

## Conclusions

This proposed assay is a reliable and powerful tool for researchers to test the compounds targeting lung Na/K-ATPase activity and, consequently, to study the impact of Na/K-ATPase in lung physiology and pathology under conditions similar to those found in the intact organ.

## Methods

### Animals

All experiments were conducted in male Swiss mice (25 – 35 g) at age 6 to 10 weeks old obtained from the Oswaldo Cruz Foundation breeding unit. The animals were lodged at 22°C with a 12 h light/dark cycle and free access to food and water. Animal housing conditions and all experimental procedures conformed to institutional regulations and were in accordance with the National Institute of Health guidelines on animal care. The institutional animal welfare committee approved all of the procedures described here under license numbers 002–08 and LW-36/10 (CEUA/FIOCRUZ).

### Preparation of oleate solutions

OA (Sigma-Aldrich, St. Louis, MO) was used to prepare a 100 mM tris-oleate solution as described in Gonçalves de Albuquerque [35]. Briefly, after weighting and water addition, sodium hydroxide was slowly added until the pH reached 13.0. The solution was maintained at 37°C and the mixture was sonicated until complete oleate solubility. Then, the pH was carefully adjusted to 7.6 with dilute hydrochloric acid*.* The working oleate solution was prepared by appropriate dilutions of the 100 mM solution with sterile saline (PBS) pH 7.5. The working oleate solutions were tested for the presence of LPS by the limulus amebocyte lysate test (LAL), which was provided by the Instituto Nacional de Controle de Qualidade em Saúde (INCQS)-Fundação Oswaldo Cruz, showing negative results.

### Experimental design and ouabain and oleic acid perfusion

Mice were divided in four groups. We used 5 to 13 animals per group. The control group was perfused with Rb^+^ free-Hank’s solution. Each animal in the second group was perfused with KCl free-Hank’s solution with Rb^+^. The third group received the same KCl free-Hank’s with Rb^+^plus ouabain, and finally the last group was perfused with KCl free-Hank’s Rb^+^plus tris-oleate.

After complete anesthesia obtained by intraperitoneal injection of 10 mg/kg of xylazin and 100 mg/Kg of ketamin dissolved in sterile saline, the thoracic cavity was opened and the left heart ventricle was cannulated using a scalpel. At the same time, right atrial appendage was cut and left open. The lung was covered with a piece of gauze immersed in saline to prevent excessive water loss by evaporation. The perfusion medium was a modified Hank’s solution (pH 7.4) containing 136.9 mM NaCl, 5.4 mM RbCl, 0.8 mM MgSO_4_, 5 mM NaHCO_3_, 0.33 mM Na_2_HPO_4_, 0.44 mM NaH_2_- PO4, 5 mM Hepes, 1.5 mM CaCl_2_, 3 mM glucose and heparin (1000 UI/l) both in the absence and in the presence of ouabain or tris-oleate. The perfusion medium was continuously oxygenated by bubbling a 95% O_2_, 5% CO_2_ gas mixture in a homemade aerating chamber. Tubing carrying this medium remained immersed in a temperature-regulated water bath adjusted to maintain the perfused lung effluent temperature at 36.5–37°C. The flow rate was 3 mL/min based on our previous results with liver [11]. The flow rate was regulated by an infusion pump (LifeMed model LF2001, Life Med Ltd., Brazil). At the end of the perfusion, physiological saline at 2–4°C was perfused at this same flow rate.

### Na/K-ATPase assay in mouse lungs based on Rb^+^ incorporation

After the rinsing procedure, the entire lung was removed, rinsed with cold saline and gently dried with filter paper. Then, 0.5 g of the lung tissue was transferred to glass tubes (250 mm × 22 mm) for digestion and 3 mL of 65% nitric acid was added. This mixture was heated in a Tecnal apparatus (model TE040125. Tecnal Ind., Brazil) at 100°C for 30 min – at this point some H_2_O_2_ drops were added to increase the speed of tissue digestion. The temperature was raised to 150°C for the next 15 min, and then to 175°C for more 15 min and finally at 200°C until the digest was completely clear. After cooling, the volume was adjusted to 15 mL with distilled water. Digested tissue samples were used to quantify Rb^+^ [11]. Briefly, for the rubidium assay we used an iCAP 6300 dual view Inductively Coupled Plasma Optical Emission Spectrometer (ICP OES) (Thermo Scientific, Cambridge, England) equipped with a Mira Mist nebulizer. Cyclonic spray chamber and a CCD detector. The operational software iTEVA 2.0 was used to data acquisition. All determinations were done in axial view and using analytical curves with nine rubidium standard solutions prepared by adequate dilution of 1,000 mg L^−1^ analytical grade Rb stock standard solution (Quimlab Química & Metrologia®, Jardim Califórnia, Jacareí, São Paulo, Brasil) until the desired concentrations using ultrapure water from a Direct 8 Milli-Q® system (Merck Millipore, Billerica, Massachusetts, EUA). The linearity of calibration curves was checked in the analytical range 0.012–0.12 μmol/L, and was not tested beyond this value. Samples having higher concentrations were diluted and re-analyzed. Results were expressed as μmol of Rb^+^ incorporated per 30 min per gram of tissue.

### Statistical analysis

Results were analyzed using “one way” ANOVA followed by Newman-Keuls (software GraphPad Prism 5.0). Values with p <0.05 were considered significant. Data are presented as mean ± SEM.

## Abbreviations

OA: Oleic acid
ARDS: Acute respiratory distress syndrome
ICP-OES: Inductively coupled plasma optical emission spectrometer.
